# Treatment of Severe Hypercapnic Respiratory Failure Caused by SARS-CoV-2 Lung Injury with ECCO_2_R Using the Hemolung Respiratory Assist System

**DOI:** 10.1155/2021/9958343

**Published:** 2021-06-29

**Authors:** Ramiro Saavedra-Romero, Francisco Paz, John M. Litell, Julia Weinkauf, Carina C. Benson, Lisa Tindell, Kari Williams

**Affiliations:** ^1^Abbott Northwestern Hospital, Minneapolis, MN 55407, USA; ^2^Minneapolis Heart Institute Foundation, Minneapolis, MN 55407, USA

## Abstract

Acute respiratory distress syndrome (ARDS) due to COVID-19 leads to a high rate of mortality in the intensive care unit (ICU). A lung-protective mechanical ventilation strategy using low tidal volumes is a cornerstone to management, but uncontrolled hypercapnia is a life-threatening consequence among severe cases. A mechanism to prevent progressive hypercapnia may offset hemodynamic instability among patients who develop hypercapnia. We present the case of a woman in her mid-60's with severe acute hypercapnic respiratory failure secondary to COVID-19 pneumonia who was successfully treated with early implementation of lung-protective ventilation facilitated by extracorporeal carbon dioxide removal (ECCO_2_R). This patient's multiple comorbid conditions included obesity, hypertension, type 2 diabetes mellitus, and hypercholesterolemia. On her fifth day of admission at the referring hospital, her worsening hypoxemia prompted endotracheal intubation during which she developed pneumothorax. She was transferred to our institution for advanced care where upon arrival, she had profound hypercapnia and respiratory acidosis. She met the criteria for treatment with an investigational ECCO_2_R device (Hemolung Respiratory Assist System) available through FDA Emergency Use Authorization. ECCO_2_R is similar to extracorporeal membrane oxygenation (ECMO) but operates at much lower blood flows (350–550 mL/min) through a smaller 15.5 French central venous catheter. Standard heparinization was provided intravenously to achieve appropriate levels of anticoagulation during ECCO_2_R therapy. Unlike ECMO, ECCO_2_R does not provide clinically meaningful oxygenation but is simpler to implement and manage. The use of ECCO_2_R successfully corrected and controlled the patient's hypercapnia and acidosis and enabled meaningful reductions in ventilator tidal volumes, respiratory rates, and mean airway pressures. The patient was weaned from ECCO_2_R after 17 days and from mechanical ventilation 10 days later. With low tidal volume ventilation facilitated by expeditious implementation of ECCO_2_R, the patient survived to discharge despite her many risk factors for a poor outcome and an extended duration of invasive mechanical ventilation.

## 1. Introduction

As the ongoing SARS-CoV-2 (COVID-19) pandemic continues to ravage the world, physicians and scientists have yet to identify a proven treatment for the deadly disease caused by the virus, especially among the critically ill. Globally, over 2 million people have died from COVID-19, compelling critical care physicians, providers, and researchers to consider improved methods for treating the unique presentations of severe acute respiratory failure associated with COVID-19. Patients who rapidly deteriorate and develop acute respiratory distress syndrome (ARDS) require life support with mechanical ventilation. Mortality in COVID-19 patients requiring mechanical ventilation, and in the severest cases, extracorporeal membrane oxygenation (ECMO), has improved over the course of 2020 but remains high, particularly for patients with certain comorbidities [1]. Early independent prognosticators of 90-day mortality from COVID-19 are older age, immunosuppression, severe obesity, diabetes, higher renal and cardiovascular SOFA score components, lower PaO_2_/FiO_2_ ratio, and a shorter time between first symptoms and ICU admission [2]. Although life sustaining, mechanical ventilation can cause further injury to lung tissue already damaged by COVID-19. Lung-protective mechanical ventilation strategies using lower tidal volumes have been shown to improve survival for patients with ARDS, but reduced tidal volumes are occasionally difficult to implement because they result in severe hypercapnia and acidosis. Both oxygen delivery and carbon dioxide elimination are required to sustain life, but occasionally, the best ventilator strategy is either insufficient to sustain life or contributes directly to severe hypercapnia, progressive acidosis, and death. Advanced devices using an extracorporeal blood circuit with hollow fiber membranes and a centrifugal pump can clear carbon dioxide independently of the lungs. Durable and clinically significant extracorporeal lung support for decarboxylation is available using low blood flow through relatively small central venous catheters. The Hemolung Respiratory Assist System is a new medical device that is currently being tested in the United States for safety and efficacy in hypercapnic respiratory failure due to emphysema (our institution is a participating study site). Recently, the U.S. Food and Drug Administration (FDA) authorized the use of the Hemolung RAS during the ongoing pandemic for patients with COVID-19 experiencing acute hypercapnic respiratory failure during standard low tidal volume mechanical ventilation. Compared to ECMO, ECCO_2_R is simpler to implement and manage and only requires extracorporeal blood flows of 350–550 mL/min and a single 15.5 French central venous catheter inserted percutaneously with a Seldinger technique. ECMO is indicated for refractory hypoxemia, but when oxygenation can be successfully managed with lung-protective mechanical ventilation, ECCO_2_R is an emerging alternative to ECMO for managing hypercapnia.

We present the case of a patient with severe ARDS secondary to COVID-19 and severe comorbidities associated with poor outcomes. Utilization of ECCO_2_R within the first day of intubation enabled implementation of lower tidal volume ventilation while maintaining arterial pH above 7.35.

Despite a protracted recovery, the patient survived and was discharged after 47 days in our ICU.

## 2. Case Description

A Hispanic woman in her mid-60's with a past medical history of obesity (BMI = 35.6), hypertension, type 2 diabetes mellitus, and hypercholesterolemia presented with a chief complaint of dyspnea and was admitted to a local hospital five days prior to being transferred to our institution.

Upon initial admission, she had a peripheral oxygen saturation of 55%, bilateral infiltrates on chest X-ray (CXR), and elevated inflammatory markers. A SARS-CoV-2 PCR test returned positive, confirming COVID-19. She was placed on noninvasive positive pressure ventilation (NIPPV) alternating with oxygen (O_2_) by high-flow nasal cannula (HFNC) at a flow of 50 LPM. Convalescent plasma, remdesivir, and tocilizumab were also administered. She did not tolerate self-proning and became increasingly dyspneic with a respiratory rate ranging from 30 to 50/min and oxygen saturation in the low 90's despite a negative fluid balance. The patient was intubated five days after admission and at that point was noted to have pharyngeal edema, which obscured laryngoscopic view and increased the level of difficulty during intubation. She developed pneumothorax after a challenging intubation with concomitant hypotension. A thoracostomy tube was placed to evacuate the pneumothorax, and the patient was treated with methylprednisolone for laryngeal edema and norepinephrine for hypotension. The patient was transferred to our institution that same day for advanced care.

Upon arrival to our ICU, her oxygen saturation by pulse oximeter (SpO_2_) was 77%, her blood pressure (BP) was 90/40 on norepinephrine at 10 mcg/min, and her initial arterial blood gas (ABG) results were pH = 7.14, PaCO_2_ = 90 mmHg, PaO_2_ = 52 mmHg, and HCO_3_ = 30 mEq/L. She had significant whole body subcutaneous crepitus, and CXR showed an inflated right lung, subcutaneous emphysema, and an appropriately positioned endotracheal tube (ETT). She was being ventilated in assist-control mode with a set tidal volume of 300 mL and FiO_2_ between 0.95 and 1.0, with a peak end-expiratory pressure (PEEP) of 16 cmH_2_O, and a mean airway pressure of 23 cmH_2_O. Her PaO_2_/FiO_2_ was <80, but we were able to stabilize her oxygen saturation. She became increasingly tachycardic and tachypneic. A repeat ABG showed further worsening of hypercapnia and respiratory acidosis. At this time, she was evaluated and met the criteria for treatment with ECCO_2_R using the Hemolung RAS. Consent to provide ECCO_2_R was requested from and received by the patient's legally authorized representative.

Heparinization was implemented per institutional protocol for extracorporeal life support, and coagulation parameters were monitored at baseline, postheparin bolus, and then every 6 hours for the duration of ECCO_2_R therapy. The left femoral vein was chosen over the jugular vein for insertion of the Hemolung 15.5 French central venous ECCO_2_R catheter for a lower risk of mechanical complication since the patient had a chest tube in place for pneumothorax and jugular vein visualization under ultrasound for placement of the catheter was obscured by subcutaneous emphysema. Upon initiation of Hemolung therapy, blood flows of 350-450 mL/min were achieved. The rate of CO_2_ removal provided by the device was controlled by changing the flow rate of room air (sweep gas flow) through the hollow fiber membranes of the Hemolung gas exchanger. Sweep flow was increased stepwise during the first 30 minutes to achieve a CO_2_ removal rate of 100 mL/min, which corresponds approximately to 40%-50% of basal metabolic CO_2_ production.

**Figure 1:**
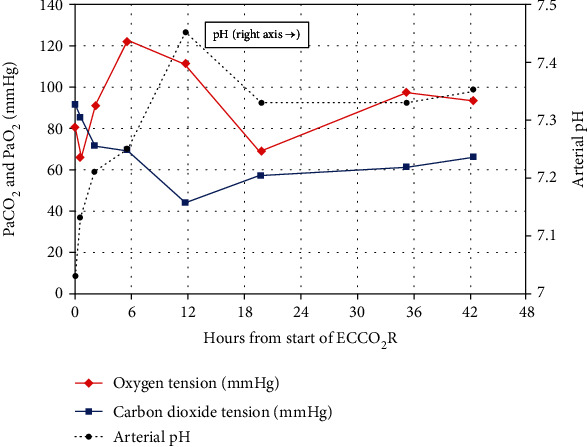
Arterial blood gases during the first 48 hours of Hemolung therapy.

**Figure 2:**
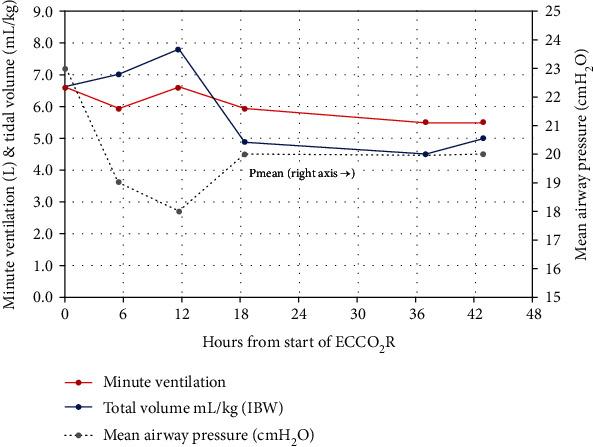
Mechanical ventilation during the first 48 hours of Hemolung therapy.

Arterial CO_2_ tension dropped from over 90 to 85 mmHg within a span of 30 minutes from the start of ECCO_2_R with concomitant improvement of arterial pH. Arterial blood gas values normalized in 24 hours, from a pH of 7.03 to 7.35 and PaCO_2_ of over 90 to 54 mmHg. Her ABG values generally remained within an acceptable range during continued ECCO_2_R therapy. Ventilator settings were maintained at PEEP of 14, rate of 26, and minute ventilation at 7.8 liters during the first 24 hours. Respiratory rate and tidal volumes were subsequently adjusted downward in a gradual manner, maintaining adequate oxygen levels and permissive hypercapnia. Tables 1 and 2 show blood gas and ventilator measures during the first 48 hours of Hemolung therapy. These measures are displayed graphically in Figures 1 and 2 showing concomitant implementation of lung-protective ventilation and control of pH, PaCO_2_, and PaO_2_.

The patient was placed in a supine and prone position alternately during treatment. The positional changes did affect circuit blood flow despite stable blood pressures and absence of femoral catheter or circuit occlusion, malposition, or kinking. Elevating the left hip in which the femoral catheter was located helped to maintain adequate blood flow through the ECCO_2_R circuit. Oxygen saturation was maintained between 90 and 100% throughout therapy. Continuous anticoagulation was given to maintain activated partial thromboplastin time (aPTT) between 45 and 90 seconds. The patient developed thrombocytopenia and anemia one week into therapy, but plasma-free hemoglobin remained less than 30 mg/dL throughout. Platelets and red blood cell transfusion were provided accordingly to maintain adequate oxygen-carrying capacity and prevent hemorrhagic complications.

The total duration of ECCO_2_R therapy was 17 days. There were no clotting or technical issues that complicated ECCO_2_R therapy nor was there any need for circuit component changes. The patient's chest tubes were removed 4 days after Hemolung decannulation. She was slowly weaned from mechanical ventilation 28 days from admission into the critical care unit with tracheostomy placement to facilitate the process. She was discharged from the hospital 47 days from admission.

## 3. Discussion

This paper describes the successful use of ECCO_2_R immediately administered with lung-protective mechanical ventilation on a COVID-19 ARDS patient with multiple risk factors for poor outcome, including pneumothorax. The Hemolung RAS allowed expeditious and adequate removal of CO_2_ with concomitant improvement and maintenance of pH within 24 hours. Carbon dioxide can be eliminated from the blood at a much higher rate than oxygen can be delivered using membrane-based extracorporeal blood gas devices due to its higher solubility and diffusivity in blood [3].

Cytokine storm, characterized as uncontrolled systemic hyperinflammation caused by cytokine excess, contributes to rapid disease progression in COVID-19 ARDS and multiorgan failure, leading to endothelial damage triggering the clotting cascade [4]. In susceptible individuals, thrombotic pulmonary vascular occlusion occurs early and progresses in a linear fashion to respiratory failure, which is a late feature of extensive vasoocclusion [5]. The patient described in this case report presented with elevated inflammatory markers, but there were no clotting or mechanical issues throughout the 17-day use of the experimental Hemolung device. Expeditious implementation of lung-protective ventilation, control of the deleterious effects of severe hypercapnia and acidosis, anticoagulation required for ECCO_2_R, and the reduced blood flows of ECCO_2_R compared to ECMO are factors that may have increased the probability of survival from severe COVID-19 ARDS.

Bleeding, catheter-associated injury or infection, thrombocytopenia, and hemolysis are known complications of ECCO_2_R. For each patient, it is important to weigh the probability and severity of these risks against the benefits of minimizing ventilator-induced lung injury and severe hypercapnia and to consistently monitor patients treated with ECCO_2_R for known complications. These complications are readily identified and quantified and, therefore, can be more effectively managed. The patient described in this report experienced thrombocytopenia and mild hemolysis over the course of 17 days which was monitored by daily blood testing. These complications were successfully managed with administration of blood products as needed and did not require discontinuation of ECCO_2_R.

Use of ECCO_2_R in conjunction with prone positioning was achieved without complications in this patient, thanks to careful management of the catheter to prevent dislodgement or damage. Adjustment of the patient's position was also necessary to maintain consistent blood flow. For this patient, obesity and generalized subcutaneous emphysema may have also contributed to blood flow variations during repositioning.

Pneumothorax, which can occur as a complication of mechanical ventilation or develop spontaneously from COVID-19 infection, has been thought to be a grave prognostic factor [6]. This case shows that the use of ECCO_2_R may have indirectly helped the patient survive what could have been a fatal complication. It is possible that decreasing mechanical power composed of the PEEP, rate, and driving pressures prevented the expansion of the patient's pneumothorax and allowed healing.

## 4. Conclusion

This case report highlights the use of ECCO_2_R to facilitate effective treatment of a patient with severe hypercapnic respiratory failure secondary to COVID-19 ARDS and multiple risk factors for death. Treatment with extracorporeal CO_2_ removal (ECCO_2_R) allowed a lung-protective ventilator management strategy with ultralow tidal volumes, minimizing the risk of ventilator-induced lung injury, attenuating severe hypercapnia and acidosis, and limiting the expansion of an existing pneumothorax. ECCO_2_R facilitates early lung-protective ventilation and control of refractory hypercapnia and can be safely utilized to increase the likelihood of survival among patients with severe COVID-19 ARDS.

## Figures and Tables

**Table 1:** Arterial blood gas measures prior to and during the first 48 hours of Hemolung therapy.

Elapsed time from start of ECCO_2_R (hrs)	Upon admit	Pre-ECCO_2_R baseline	First 48 hours of ECCO_2_R						
	3 hrs prior to ECCO_2_R	2 hrs prior to ECCO_2_R	0.6	2.1	5.5	11.7	19.7	35.3	42.4
pH	7.14	7.03	7.13	7.21	7.25	7.45	7.33	7.33	7.35
PaCO_2_ (mmHg)	90	>90	85	71	69	44	57	61	66
PaO_2_ (mmHg)	52	80	66	91	122	111	68	97	93
HCO_3_ (mEq/L)	30	29	28	28	30	31	30	32	36
SaO_2_ (%)	77	94	93	98	100	100	96	98	98

**Table 2:** Mechanical ventilation parameters prior to and during the first 48 hours of Hemolung therapy.

Elapsed time from start of ECCO_2_R (hrs)	Upon admit	First 48 hours of ECCO_2_R					
	—	5.5	11.7	18.5	37.0	42.9	63.2
Ventilation mode	A/C	A/C	A/C	A/C	A/C	A/C	A/C
Respiratory rate—actual (/min)	22	26	26	18	18	20	20
Tidal volume—set (mL)	300	270	300	270	250	250	250
Tidal volume/kg IBW (mL/kg)	6.6	5.9	6.6	5.9	5.5	5.5	5.5
Minute ventilation (L)	6.6	7.0	7.8	4.9	4.5	5.0	5.0
Peak end-expiratory pressure (cmH_2_O)	16		14	12	14	14	12
Peak inspiratory pressure (cmH_2_O)	34	29	31	30	33	32	26
Mean airway pressure (cmH_2_O)	23	19	18	20	20	20	19
Inspired oxygen fraction (%)	98		98	88	89	79	79

## Data Availability

Raw data were generated at Abbott Northwestern Hospital. Derived data supporting the findings of this study are available from the corresponding author (RSR) on request.
